# A metagenome-derived thermostable β-glucanase with an unusual module architecture which defines the new glycoside hydrolase family GH148

**DOI:** 10.1038/s41598-017-16839-8

**Published:** 2017-12-11

**Authors:** Angel Angelov, Vu Thuy Trang Pham, Maria Übelacker, Silja Brady, Benedikt Leis, Nicole Pill, Judith Brolle, Matthias Mechelke, Matthias Moerch, Bernard Henrissat, Wolfgang Liebl

**Affiliations:** 1 Department of Microbiology, School of Life Sciences Weihenstephan, Technical University of Munich, Freising-Weihenstephan, Germany; 2 Department of Genomic and Applied Microbiology and Göttingen Genomics Laboratory, Georg-August University Göttingen, Göttingen, Germany; 3 Architecture et Function des Macromolécules Biologiques, CNRS, Aix-Marseille University, Marseille, France

## Abstract

The discovery of novel and robust enzymes for the breakdown of plant biomass bears tremendous potential for the development of sustainable production processes in the rapidly evolving new bioeconomy. By functional screening of a metagenomic library from a volcano soil sample a novel thermostable endo-β-glucanase (EngU) which is unusual with regard to its module architecture and cleavage specificity was identified. Various recombinant EngU variants were characterized. Assignment of EngU to an existing glycoside hydrolase (GH) family was not possible. Two regions of EngU showed weak sequence similarity to proteins of the GH clan GH-A, and acidic residues crucial for catalytic activity of EngU were identified by mutation. Unusual, a carbohydrate-binding module (CBM4) which displayed binding affinity for β-glucan, lichenin and carboxymethyl-cellulose was found as an insertion between these two regions. EngU hydrolyzed β-1,4 linkages in carboxymethyl-cellulose, but displayed its highest activity with mixed linkage (β-1,3-/β-1,4-) glucans such as barley β-glucan and lichenin, where in contrast to characterized lichenases cleavage occurred predominantly at the β-1,3 linkages of C4-substituted glucose residues. EngU and numerous related enzymes with previously unknown function represent a new GH family of biomass-degrading enzymes within the GH-A clan. The name assigned to the new GH family is GH148.

## Introduction

Based on amino acid sequence and in consequence also structural similarity, glycoside hydrolase (GH) enzymes are grouped into GH families, as comprehensively documented in the carbohydrate-active enzymes (CAZy) database (http://www.cazy.org/). The related enzymes within a GH family share structural features and the basic catalytic mechanism (retaining or inverting), but some GH families display a remarkably broad diversity of substrate specificities among their members. GHs are often modular enzymes which, in particular if they are involved in the degradation of insoluble polysaccharides such as cellulose, can contain carbohydrate-binding modules (CBMs) in addition to the catalytic module(s).

With the ongoing transition from traditional chemical production to more sustainable and environment-friendly processes, the biotechnology-related industries face a significant demand for suited enzymes. Different branches of the industry such as the chemical, biofuel, food, feed and pharmaceutical industry have a large interest in hydrolases that cleave the glycosidic bonds of plant-derived polysaccharides such as mixed-linkage β-glucans, cellulose, or xylan^1,2^. The degradation of these polysaccharides usually requires the action of multiple enzyme activities^3^. In addition to functionality, high thermostability and other properties contributing to a robust behavior of these proteins under harsh process conditions are demanded from the industrial perspective^4–6^.

In the course of searching for novel enzymes for industrial applications, metagenomic analysis can overcome the limitations of culture-dependent methods and can facilitate the discovery of enzymes from uncharacterized microorganisms^7–12^. In combination with advanced library preparation, next-generation sequencing and high throughput screening techniques, metagenomic approaches are evolving to exploit the enormous potential of nature’s microbiological diversity^13–15^. Although sequence-based screening is easy and time-saving, the often laborious functional approaches have the advantage that they are sequence-independent and can uncover completely unknown enzymes or reveal new activities^16–18^. In the last few years, functional screening has helped to discover novel enzymes from various environmental starting materials such as from gut microbiome, biogas plant or soil samples^18–21^.

In this study, we describe a novel thermostable endoglucanase (EngU) that was found by functional screening of a metagenomic library from a volcano site sample from the Avachinsky crater in Siberia, Russia^22^. Sequence analysis of EngU revealed regions with limited local amino acid sequence similarity to glycoside hydrolase enzymes of the GH-A clan. However, the enzyme could not be assigned to an existing GH family. Local sequence similarity exists between EngU and GH42, comprising enzymes with β-galactosidase or α-l-arabinopyranosidase activity^23^, but EngU showed a completely different activity profile cleaving glucan polymers like barley β-glucan, lichenin and carboxymethyl cellulose. In addition to its unexpected substrate preference, EngU revealed a novel modular structure that warranted further inspection of this currently uncharacterized enzyme type.

## Results

### Metagenomic library screening

In a recent metagenomics study with samples originating from the Avachinsky Crater (Kamchatka Peninsula, Russia), several fosmid clones were identified which showed activity on 4-methylumbelliferyl-β-d-cellobioside (4-MUC) as well as on carboxymethyl-cellulose (CMC) agar plates^22^. One particular clone, FosK48 H3, attracted our interest because a putative β-galactosidase gene ortholog was the only glycosidase-encoding candidate gene identified within the 32.99 kbp metagenomic insert sequence. The corresponding candidate protein, named EngU (for endo-glucanase), was subjected to further investigation.

### Domain organization of EngU and expression of *engU* fragments

**Figure 1:**
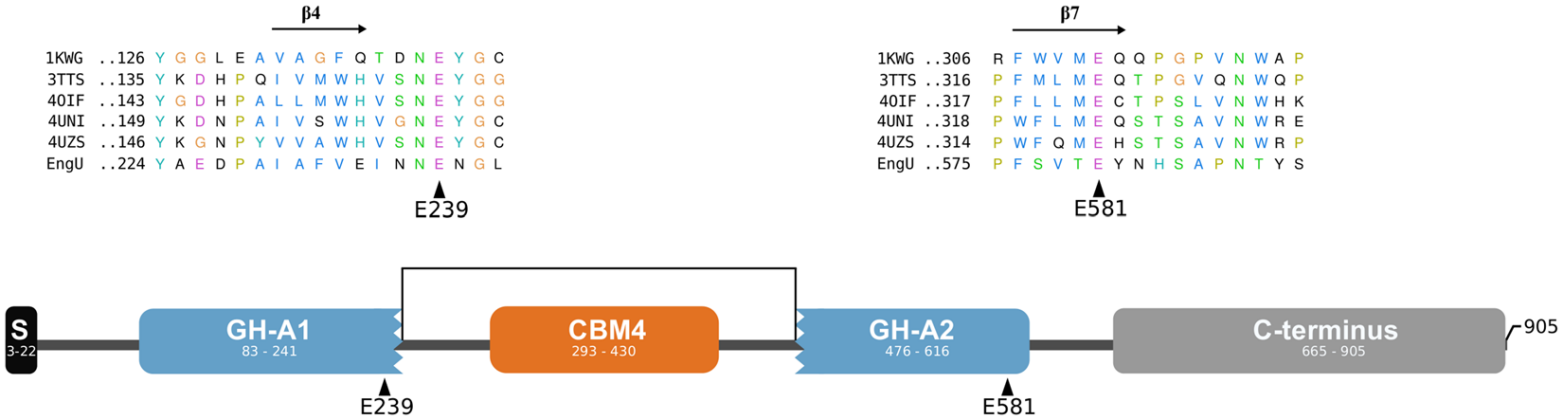
Scheme of the modular architecture of EngU. Segments of the 905 residue EngU sequence with distant similarity to parts of Pfam families are colored in blue (GH42) or orange (CBM4_9). The coordinates of the regions with similarity are indicated as numbers within the colored boxes. The N-terminal signal peptide of EngU is drawn as a black box. The C-terminus (grey) of EngU showed no detectable similarity to known Pfam families. Alignments of EngU with representative GH42 sequences around the catalytic residues are shown above the scheme. The proteins used in the alignment are 1KWG (*Thermus thermophilus* A4 β-galactosidase), 3TTS (*Bacillus circulans* β-galactosidase), 4OIF (*Geobacillus stearothermophilus* β-galactosidase), 4UNI (*Bifidobacterium animalis* subsp. *lactis* β-(1,6)-galactosidase) and 4UZS (*Bifidobacterium bifidum* β-galactosidase).

**Figure 2:**
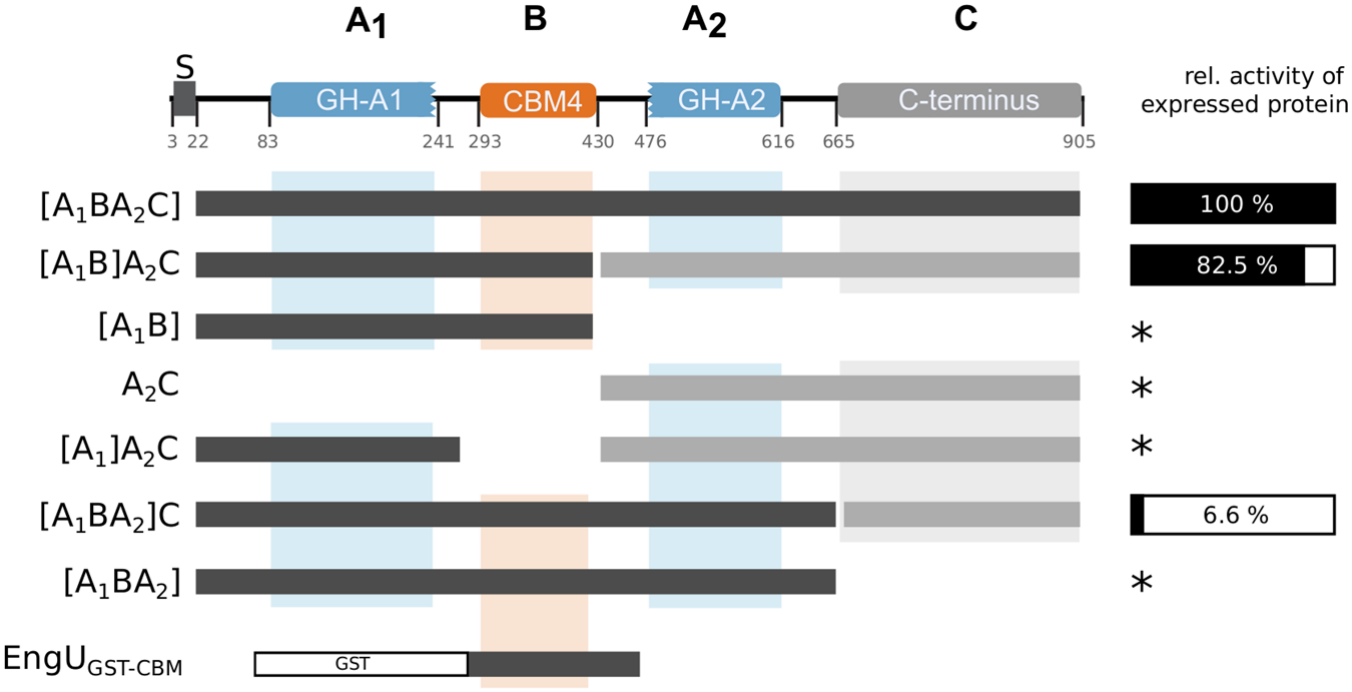
Modular composition of EngU and an overview of EngU variants cloned in pETDuet. EngU fragments cloned in the first multiple cloning site (MCS1) are dark colored and written in square brackets while fragments in the second MCS2 are drawn as brighter bars. The pETDuet constructs were named according to the annotated parts of EngU, where A_1_ includes GH-A1, B is the CBM, A_2_ is GH_A2 and C includes the C-terminus with unknown function. The activities of successfully expressed proteins with the substrate barley β-glucan (percent relative to the full-length enzyme) are shown on the right. Derivatives marked with a star (*) were expressed as insoluble, inactive proteins.

The domain architecture of the EngU protein sequence was analyzed by searching against the Pfam and Superfamily databases and, in order to increase sensitivity, against a Pfam subset which contained only models of glycoside hydrolases and carbohydrate binding modules. These searches revealed an unusual mosaic-like module arrangement of EngU (Fig. 1). Two non-contiguous segments within the 905 residue EngU primary structure displayed partial similarity to the Pfam seed representing GH family 42 and to the β-glycanases family in the Superfamily database (family 51487), which suggested EngU belongs to the GH-A clan of glycoside hydrolases (Fig. 1). A GH42-like region of EngU (GH-A1, also designated as A_1_ in the names of expression vector constructs mentioned below and in Fig. 2) which however was insufficient to represent a functional GH42 enzyme reaches from position 83 to 241. A more sensitive sequence analysis, including the manual inspection of sequence stretches with possible similarity to conserved regions surrounding catalytic residues of clan GH-A members revealed a second region (GH-A2, also designated as A_2_) between residues 476 to 616 of EngU. A carbohydrate-binding module CBM4 (residues 293 to 430) was found to be inserted between the two β-glycanase parts which results in a large distance of the key catalytic residues along the primary structure of EngU (see site-directed mutagenesis experiments below). In contrast to many modular glycoside hydrolases where catalytic and substrate-binding modules are connected by linker regions, no such linkers could be detected between the catalytic module and CBM4 parts of EngU. The C-terminus of the protein did not exhibit detectable similarity to known protein families. The presence of an N-terminal signal peptide was found with the SignalP algorithm (amino acid positions 3–22).

**Figure 3:**
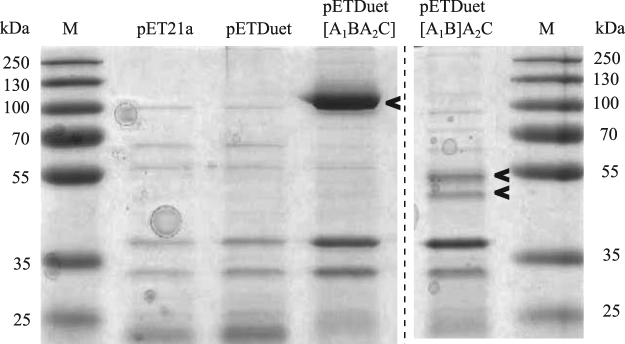
SDS-PAGE of heat-treated extracts from *E*. *coli* clones expressing EngU variants. M is the protein molecular weight marker, 10 µg protein per lane were loaded. The names of EngU derivatives are as in Fig. 2. The *in silico* predicted molecular weights are 100.2 kDa for the full-length EngU (containing parts A_1_BA_2_C, or amino acid positions 24 to 905), 46.9 kDa for A_1_B (containing amino acid positions 24 to 432 of EngU) and 54.5 kDa for A_2_C (containing amino acid positions 421 to 905 of EngU). The coding regions for parts written in square brackets were cloned in the first MCS, coding regions for C-terminally located enzyme parts were inserted into the second MCS of the pETDuet vector. This figure was assembled from two SDS PAGE gels.

To dissect the functions of each domain, several expression constructs were made by classical cloning methods or Gibson Assembly (summarized in Fig. 2). DNA segments encoding truncated versions and differently combined domains of EngU were amplified with primers listed in the supplementary material (Supplementary Table S1) and inserted into the pETDuet vector. The names of the plasmids used in the following specify the encoded EngU regions, where the fragment written in square brackets is inserted in the first multiple cloning site (MCS) of pETDuet. For example, pETDuet-[A_1_BA_2_C] carries the whole protein sequence without the signal peptide (residues 23–905) in the first MCS and in pETDuet-[A_1_B]A_2_C the *engU* sequence was split after the CBM and the two parts were cloned into the same vector using both of its MCSs. This separation leads to the expression of EngU as two separate polypeptide chains in the same cell. We also generated vectors for expression of only one of the two GH-A parts, pETDuet-[A_1_B] and pETDuet-A_2_C. Further expression vectors were designed, some lacking the regions encoding the CBM or the C-terminus, and some expressing the C-terminus separately from the other parts of the enzyme to investigate the role of the C-terminus for EngU. Some but not all expression strains yielded soluble or active enzyme (Fig. 2). SDS-PAGE analysis after protein expression with pETDuet-[A_1_B]A_2_C revealed two bands of soluble and heat-stable proteins whose molecular masses corresponded to the two expected polypeptide chains expressed from the two MCSs of this expression plasmid (predicted molecular masses: 46.9 kDa for A_1_B and 54.5 kDa for A_2_C) (Fig. 3). The two separately expressed parts complemented each other and reconstituted glucanase activity in the cell, which was not observed if only one part was expressed alone (either pETDuet-[A_1_B] or pETDuet-A_2_C). Co-expression of the two separate enzyme halves resulted in merely a slight reduction of the activity (82.5%) of the crude cell extract compared to the strain expressing full-length EngU (100%). While truncation of the C-terminal domain did not yield a functional enzyme, another dual-polypeptide expression clone, carrying pETDuet-[A_1_BA_2_]C, in which the EngU C-terminus was co-expressed together with the remainder of EngU yielded only 6.6% relative activity, indicating a possible role of the C-terminus for activity and/or stability of EngU.

The construct EngU_GST-CBM_, designed to study the properties of the CBM module of EngU (amino acid positions 292–473) as a N-terminal glutathione S-transferase (GST) fusion protein (see Fig. 2), could also be expressed as a soluble protein and its binding properties to different polysaccharides could be investigated.

### Enzymatic characterization of EngU and binding properties of EngU_GST-CBM_

Full-length EngU protein could be obtained by pET-based expression in *E*. *coli*. Due to a higher expression level, a truncated enzyme lacking 20 amino acid residues from the N-terminus was used for further expression, purification and characterization of EngU. Heat treatment of crude extract, removal of the precipitated host proteins and cationic exchange chromatography yielded a purified recombinant protein of approx. 100 kDa which is in accordance with the predicted molecular weight of 100.2 kDa (Fig. 3 and Table 1). Sufficiently pure preparations of EngU_GST-CBM_ could also be obtained, using a combination of affinity and size-exclusion chromatography.

**Table 1:** Purification of recombinant EngU. Activity was measured by the DNS assay with 0.5% barley β-glucan as the substrate in 50 mM McIlvaine buffer pH 6.0 after incubation for 10 min at 80 °C.

Fraction	Vol [mL]	Protein [mg/mL]	Spec. Activity [U/mg]	Purification factor	Yield [%]
Crude extract	25	8.5	2	1	100
Heat treated crude extract	22	0.9	8.3	4.2	41
Elution Source 15S	3	0.2	132.9	67.5	19.8

**Figure 4:**
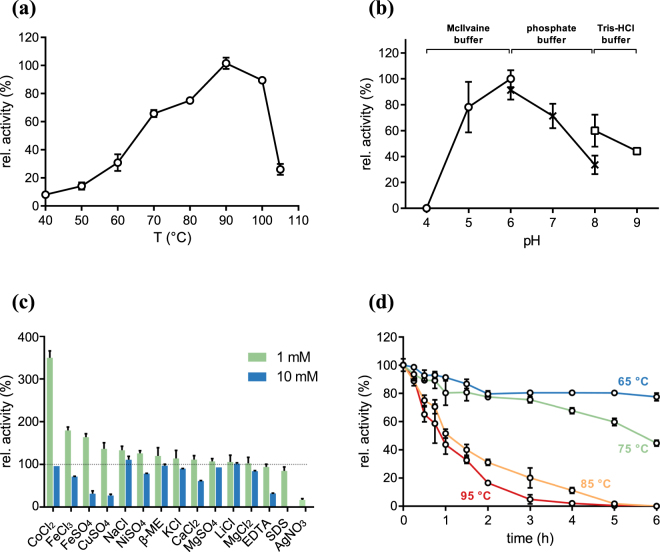
Influence of temperature, pH and various additives on EngU activity. All assays were performed in triplicate, the error bars represent standard deviations. (**a**) Temperature-*vs-*activity profile. The relative activity of EngU (0.06 mg/ml) with barley β-glucan was measured at the indicated temperatures in McIlvaine buffer, pH 6 and 25 min incubation time. (**b**) Dependence of activity on the pH. Incubations were performed with 0.06 mg/mL purified protein at 90 °C for 45 min in McIlvaine (pH 4–6), phosphate buffer (pH 6–8) and Tris-HCl buffer (pH 8–9). (**c**) Influence of metal ions, SDS, EDTA and β-mercaptoethanol (β-ME) on the activity of EngU with barley β-glucan. Incubations were performed with 0.06 mg/ml EngU at 90 °C for 12 min in McIlvaine buffer, pH 6. The activity without additions is set to 100%. (**d**) Kinetics of thermoinactivation of EngU. After incubation of recombinant EngU (0.2 mg/mL) in 50 mM McIlvaine buffer pH 6 for the indicated time, the remaining activity was measured at 80 °C in 50 mM McIlvaine buffer pH 6 for 20 min, using barley β-glucan as the substrate. The activity at t = 0 was set to 100%.

EngU was most active at temperatures around 90 °C with a pH optimum at 6.0 under our standard assay conditions (10 min incubation, Fig. 4). The enzyme retained most of its activity over several hours at temperatures up to 75 °C. The highest activity was measured with barley β-glucan (132.9 U/mg protein) and the structurally similar substrate lichenin (80% of the activity with β-glucan). The hydrolysis of soluble cellulose derivatives such as CMC and HEC was relatively weak, with activities of less than 10% compared to β-glucan cleavage. No degradation of 5-bromo-4-chloro-3-indoxyl-β-D-galactopyranoside (X-gal) and no release of reducing sugar from the β-1,3-glucans pachyman and laminarin, and from microcrystalline cellulose could be measured (Table 2). The influence of the anionic surfactant SDS, the reducing agent β-mercaptoethanol, and the chelating agent EDTA on the activity of EngU was also investigated. In general, concentrations of 1 mM of any of these compounds had no significant impact. However, 10 mM SDS resulted in the complete loss of activity. In the presence of 10 mM EDTA, the activity was reduced to 33%, indicating the possible need for divalent cations for enzymatic activity or stability. Supplementation with ferrous and ferric ions at 1 mM had a positive effect on EngU activity. Notably, 1 mM cobalt chloride addition enhanced the EngU activity 3.5-fold compared to assay conditions without additional cofactor addition (Fig. 4c).

**Table 2:** Substrate spectrum of EngU. Hydrolytic activities of EngU were determined at 80 °C in McIlvaine buffer at pH 5.5.

**Substrate**	**Moiety**	**glycosidic linkage**	**Relative activity (%)**
β-Glucan (barley)	β-D-glucopyranose	β-1,3; β-1,4 linear	100
Lichenin (*C*. *islandica*)	β-D-glucopyranose	β-1,3; β-1,4 linear	93
Carboxymethyl-cellulose	β-D-glucopyranose with carboxylmethylated hydroxyl groups	β-1,4 linear	23
Hydroxyethylcellulose	β-D-glucopyranose	β-1,4 linear	2
Xyloglucan	β-D-glucopyranose, α-D-xylopyranose side chains	β-1,4 linear	<1
*Substrates showing no activity (below the detection limit of the DNS or the bromo-chloro-indoxyl assay) with EngU after overnight incubation*			
Pachyman (*P*. *cocos*)	β-D-glucopyranose	β-1,3	
Zymosan (*C*. *cerevisae*)	β-D-glucopyranose	β-1,3 linear, protein complex	
Curdlan (*A*. *faecalis*)	β-D-glucopyranose	β-1,3 linear	
Auxoferm (*S*. *cerevisae*)	β-D-glucopyranose	β-1,3	
Laminarin (*L*. *digitata*)	β-D-glucopyranose	β-1,3; 1,6 linear	
Microcrystalline cellulose	β-D-glucopyranose	β-1,4 crystaline structure	
Arabinoxylan	arabinose: 36%, xylose: 51%, glucose: 6.5%, mannose: 4.4%, galactose: 1.6%	β-1,4; β-1,3 branched	
Dextran	α-D-glucopyranose	α-1,6; α-1,4; α-1,3;	
Galactan	galactose: 87%, arabinose: 5%, rhamnose: 1%, xylose: 1%, galacturonic acid: 5%, other sugars	β-1,4; β-1,3 branched	
Galactomannan	β-D-mannopyranose, β-D-galactopyranose side chains (62:38)	β-D-1,4 and side chain α-D-1,6 branched	
Inulin (dahlia bulb)	fructose, glucose	β-2,1 linear	
Laminarin	β-D-glucopyranose	β-D-1,3 with β-D-1,6 branches	
Levan (*E*. *herbicola*)	α-D-fructose	α-D-2,6 linear	
Mannan	mannose: 98%, galactose: ca. 1%	β-D-1,4 linear	
Mannan (Ivory nut)	mannose: 99%, arabinose, xylose: traces	β-D-1,4 linear	
“Pectic galactan” (Lupine)	galactose: 74%, arabinose: 17%, rhamnose: 3%, xylose: 1%, galacturonic acid: 5%, glucose (trace)	β-D-1,4 linear	
“Pectic galactan” (potato)	galactose: 82%, arabinose: 6%, rhamnose: 3%, galacturonic acid: 9%	β-D-1,4 linear	
Polygalacturonic acid	α-D-galacturonic acid	α-D-1,4 linear	
Polyoses (Hemicellulose)	xylose, arabinose, glucose, mannose, galactose		
Pullulan	maltotriose	Glu: α-D-1,4/ Maltotriose: α-D-1,6 linear	
Rhamnogalacturonan I	α-D-Galacturonic acid, α-L-rhamnopyranose	GalUA: α-D-1,6/ Rha: α-L-1,2 branched	
Sinistrin	β-D-Fructopyranose	β-D-1,2 and 1,6 branched	
X-Gal	5-Bromo-4-chloro-3-indoxyl-β-D-galactopyranoside		
Xylan (Birch wood)	β-D-Xylopyranose	β-D-1,4 branched	
Xylan (Oat spelt)	β-D-Xylopyranose	β-D-1,4 branched	
Xylan (Larch wood)	β-D-Xylopyranose	β-D-1,4 branched	

**Figure 5:**
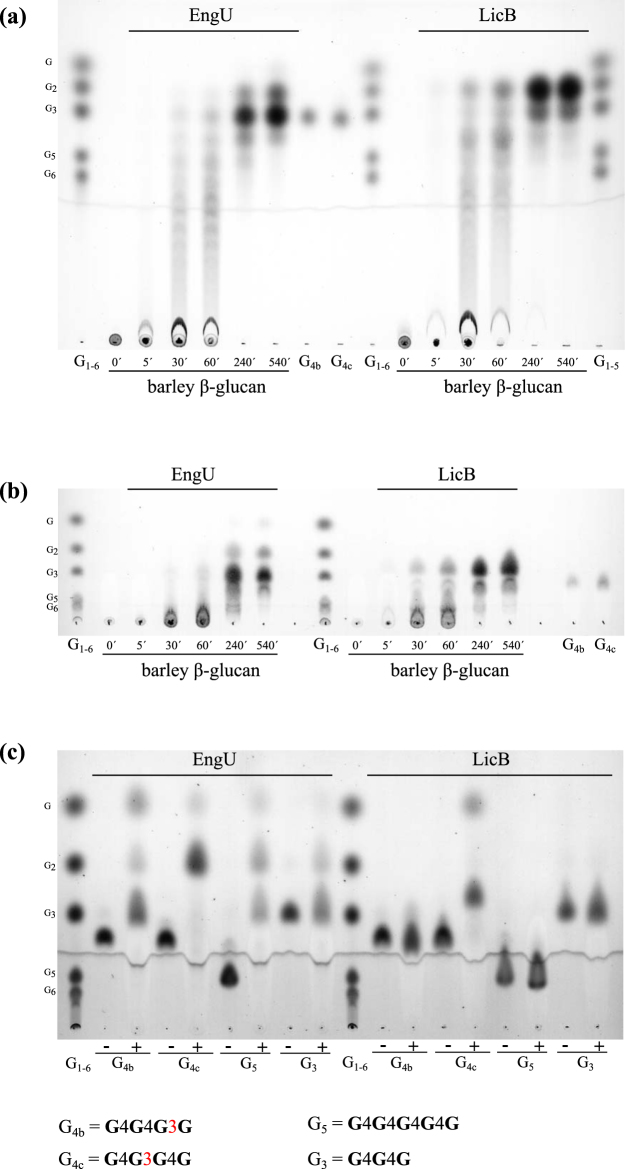
Thin layer chromatography analysis of barley β-glucan and oligosaccharide degradation products by purified EngU and *Clostridium thermocellum* LicB. The samples were incubated in 50 mM McIlvaine buffer pH 6 at 80 °C for EngU and 65 °C for LicB. The reactions contained 15 µg/ml EngU or 7 µg/ml LicB and 0.5% barley β-glucan or 0.1% of the defined oligosaccharides cellopentaose (G_5_), cellotriose (G_3_), or the mixed-linkage glucotetraoses G_4__b_ (G4G4G3G) and G_4__c_ (G4G3G4G). The cellooligosaccharide standard used (G_1–6_) is a mixture of 0.1% each of glucose, cellobiose, -triose, -pentaose and -hexaose. The mobile phase was butanol:ethanol:water (in a volume ratio of 5:5:4) in (**a)** and acetonitrile:water (in a volume ratio of 8:2) in (**b)** and (**c).** While LicB preferentially cleaves β-1,4 linkages of glucose moieties that are substituted at C3, EngU preferentially cleaves β-1,3 linkages of glucose moieties that are substituted at C4 (see Fig. 6), resulting in mainly β-glucooligosaccharides with a β-1,3 linkage (LicB) or mainly cellotriose (EngU) from mixed-linkage β-glucan (TLC sheets A and B).

**Figure 6:**
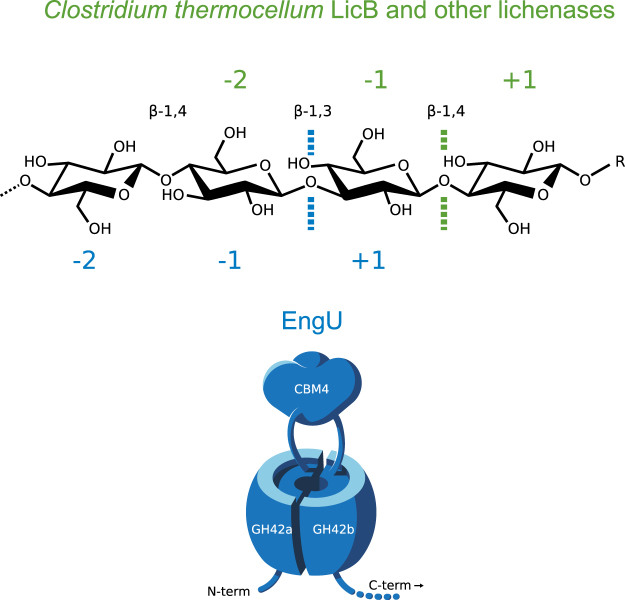
Schematic model and bond cleavage preference of EngU. The EngU model shows the presumed modular composition of the enzyme with a TIM barrel assembled from the two half-barrels GH-A1 and GH-A2. In the β-glucan structure section shown, the main glycosidic bond cleavage preference of EngU is indicated as a blue line, while the typical bond cleavage preference of other lichenases is shown in green.

For a deeper insight into the bond cleavage specificity of EngU we analyzed by TLC and HPAEC-PAD the hydrolysis products liberated from barley β-glucan, two different mixed-linkage β-1,3/ β-1,4-glucotetraose substrates (G_4b_ = G4G4G3G, and G_4c_ = G4G3G4G), as well as cellopentaose (G_5_) and cellotriose (G_3_) (Fig. 5 and Supplementary Figure 3). These experiments were performed also with the purified mixed-linkage β-glucanase (lichenase) LicB from *Clostridium thermocellum* (UniProt accession number Q84C00), for which the cleavage specificity with β-glucan is well known^24,25^, in order to aid the interpretation of the results with EngU. The first detectable intermediate products of β-glucan cleavage by EngU were oligosaccharides with degree of polymerization (DP) ranging from 6 to 9 hexose moieties and larger, suggesting an *endo*-mechanism for β-glucan degradation (Fig. 5a). The endo-mode is corroborated by the activity on CMC and HEC. After prolonged incubation, the major final product had a mobility similar to cellotriose (G_3_) of the G_1–6_ standard. However, the end-products of β-glucan cleavage by EngU and LicB differed in their mobility in TLC and HPAEC analysis. This difference can be explained by the presence or absence of a β-1,3 linkage in the products (Fig. 5b and Supplementary Figure 3). LicB cleaves predominantly β-1,4 linkages of glucose residues which are themselves substituted at C-3, in other words cleavage occurs at a β-1,4 following a β-1,3 linkage in mixed-linkage β-glucans (Fig. 6). The end products of β-glucan hydrolysis by LicB would in this way contain one β-1,3 bond, and the presence of this bond and even its position leads to an altered mobility of the products in both TLC and HPAEC (faster mobility in TLC and later elution in HPAEC). In contrast to LicB, and mirroring its cleavage specificity, EngU appears to cleave mainly β-1,3 linkages of glucose residues which are themselves substituted at C-4, leading to cellotriose (G_3_ = G4G4G) as the main product of β-glucan hydrolysis. This marked difference in the cleavage specificity of the two enzymes was further confirmed by reactions with short glucose oligomers with defined structures, e.g. the exclusively β-1,4-linked gluco-(cello-)oligosaccharides G_5_ (G4G4G4G4G) and G_3_ (G4G4G) or the mixed-linkage β-1,3/β-1,4-glucooligosaccharides G_4__b_ (G4G4G3G), G_4__c_ (G4G3G4G). In full agreement with what was proposed above to explain the product pattern obtained from β-glucan cleavage, EngU was able to hydrolyze the G_4__b_ and G_4__c_ substrates, apparently cleaving at their β-1,3 linkages (and weakly also β-1,4 linkages), while LicB hydrolyzed G_4__c_ only, obviously due to the presence of a β-1,4-linked and C-3 substituted glucose residue in this substrate (Fig. 5c). After extended incubation, cellopentaose, and to a lesser extent cellotriose were also hydrolyzed by EngU, which is in line with its observed weak activity with β-1,4-cellulosic substrates such as CMC and HEC (Table 2). On the other hand, purely β-1,3-linked glucans like zymosan and curdlan or β-1,3-1,6 glucans like laminarin were not substrates for EngU (Table 2). EngU was not able to cleave lactose, a typical substrate of GH42 enzymes.

The functionality and the polysaccharide binding characteristics of the EngU CBM4 module was investigated in two ways: (i) by measuring the depletion of EngU_GST-CBM_ fusions in cleared supernatants after incubation with insoluble substrates and (ii) by affinity gel electrophoresis which measures the retardation of the purified fusion protein in a native PAGE gel containing the soluble binding substrates (Table 3 and Supplementary Figure 4). In affinity gel electrophoresis, EngU_GST-CBM_ was strongly retarded by β-glucan and to a lesser extent by lichenin and CMC, while no retardation was observed with the β-1,3/β-1,6-linked glucan laminarin. With the insoluble cellulose substrates, binding of EngU_GST-CBM_ was strongest with amorphous cellulose (phosphoric acid swollen cellulose, PASC) and decreased with increasing crystallinity of the cellulose preparations. Insoluble β-1,3-glucan polymers like pachyman or Auxoferm were not binding substrates for EngU_GST-CBM_. As a control to exclude that the GST part of the fusion protein was responsible for binding of EngU_GST-CBM_ the binding properties of GST alone was also assessed in both the gel shift assay and the batch assay. No binding of GST to any of the tested substrates was measured.

**Table 3:** Summary of the binding properties of purified EngU_GST-CBM_ and GST (used as control) to soluble and insoluble substrates (see also Supplementary Figure 4).

Substrate	GST		EngU_GST-CBM_	
*Soluble substrates (gel shift assays)*	*r/r0*	*K*r	*r/r0*	*K*r
β-glucan	1.03	>10	0.16	0.19
Lichenin	1.10	>10	0.30	0.43
CMC	1.15	>10	0.34	0.52
Laminarin	1.08	>10	1.07	>10
*Insoluble substrates (batch assays)*	*Level of binding*			
	**GST**		**EngU**_**GST-CBM**_	
PASC	No binding		Strong	
Avicel	No binding		Weak	
CF1 cellulose	No binding		No binding	
Xylan (birch wood)	No binding		Weak	
Pachyman	No binding		No binding	
Auxoferm	No binding		No binding	

*K*r, retardation coefficient (mg × ml^−1^). *r*_0_ and *r*, relative migration distance without and with substrate (at 1 mg × ml^−1^), respectively. The batch assays for insoluble substrates were performed with 100 µg protein and 50 mg substrate.

### Site directed mutagenesis

EngU was aligned with sequence profiles from the GH-A clan (families GH5 and GH42) as well as with the CBM4 Pfam family using the program *hmmscan* from the HMMER 3.1 package^26^. Based on this alignment, acidic residues possibly involved in catalysis were identified and substituted with alanine in order to investigate their role in the ability of EngU to cleave polymeric substrates. All mutants and their activities towards CMC are listed in Table 4. In particular, short sequence regions surrounding the two conserved catalytic glutamate residues in the Pfam GH42 model (E136 and E295) could be aligned to E239 and E581 in EngU (Fig. 1). E239A (predicted acid/base catalytic residue) and E581A (predicted nucleophile) mutants showed a complete loss of activity compared to the wild type protein. Instead of being located in a contiguous catalytic module as is usual in most GH enzymes, the two key catalytic amino acid residues of EngU are obviously located in two halves of the non-contiguous catalytic module separated by the 131 residue carbohydrate-binding module (residues 294 to 425).

**Table 4:** Site-directed mutants and their relative activity compared to wild type EngU. Hydrolytic activities towards 2% carboxymethyl-cellulose (CMC) were determined using the DNS assay with heat-treated, cleared preparations of EngU and mutants.

Protein	Relative CMCase activity
EngU wt	100%
EngU D123A	3%
EngU D151A	104%
EngU E183A	124%
EngU E235A	28%
EngU E239A	0%
EngU E581A	0%
EngU E593A	51%
EngU D605A	32%

## Discussion

Using a function-based metagenomic screening strategy, the gene encoding a novel extremely thermostable glycoside hydrolase, an endo-β-glucanase termed EngU, was identified on the insert of a recombinant fosmid from a DNA library from the Siberian Avachinsky crater. EngU represents a putative GH-A clan member which could not be assigned to an existing GH family. A recent BLAST search using EngU as the query sequence exposed numerous hits which showed similarity along the full length of EngU. The best-scoring putative EngU homologs with up to 48% amino acid sequence identity were hypothetical proteins from uncultured bacteria (*Lentisphaerae*, *Latescibacteria*) and from uncultured archaea (*Ignisphaera aggregans*, candidatus *Bathyarchaeota* archaeon B24) but homologs could be detected also in γ- and β-Proteobacteria (genera *Cellvibrio* and *Paludibacterium*). Interestingly, most of the hits with full-length similarity to EngU were from single-cell sequencing or metagenomics data.

EngU did not show sufficient and clear similarity for inclusion into an existing GH family from the CAZy database. Based on the very limited similarity to known CAZy members, the distinct cleavage pattern of the enzyme (see below), and the relatively broad phylogenetic distribution of homologs, we propose that the catalytic domain of EngU (part A1 + A2) is the founding member of a new GH family, GH148. Like all CAZy families, this new family should be defined solely by the sequence of its catalytic domain. To determine which other sequences can be included in family GH148, we constructed an artificial sequence corresponding to EngU without the CBM4 part and used it for a Blast search against the non-redundant protein sequence databank of the NCBI. This sequence had significant hits with hundreds of sequences, 80 of which are present also in GenBank as finished entries and now displayed in the CAZy database as members of GH148 (http://www.cazy.org/GH148.html).

At residues 293–430 in the EngU protein, a CBM4 module separates GH-A1 from GHA-2 and thus splits the proposed new GH module in two. Other examples of GH polypeptides with insertions in the catalytic module can be found in sequence databases (for instance in GH10: GenBank ADD61481.1 or CCO21036.1) but further biochemical analysis is lacking. The CBM4 type of CBMs is often found in bacterial enzymes which can attach to xylan, β-1,3 and mixed-linkage glucans, and amorphous cellulose^27^. In known NMR or crystal structures of CBM4 representatives the N- and C-terminal ends of the CBM supersecondary structures are typically close to each other (PDB entries: 1ULO, CBM4 from *Cellulomonas fimi* CenC; 1CX1, CBM4 from *Cellulomonas fimi* EngC; 1K42, 1K45, CBM4–2 from *Rhodothermus marinus* xylanase; 3K4Z, CBM4 from *Clostridium thermocellum* CbhA; 3P6B, CBM4 from *Clostridium thermocellum* CelK; 1GUI, CBM4 from *Thermotoga maritima* Lam16A)^27–32^. It seems as if the CBM module-encoding sequence was inserted into a formerly contiguous GH module gene by an evolutionary incident leading to the ancestral gene of the *engU*-similar genes from numerous yet uncultivated bacteria.

By expressing the CBM4 part of EngU as a GST-fusion protein, we could show that the CBM4 module is functional, displaying strong binding to soluble (barley β-glucan) as well as insoluble (amorphous cellulose) substrates. In line with the substrate preference of the catalytic module of EngU (see below), the carbohydrate binding module did not bind to β-1,3- or β-1,3-1,6 glucan polymers like laminarin, pachyman and auxoferm. CBM4 family proteins belong to the type B glycan chain binding CBMs which have binding site topologies suited to interact with individual glycan chains, rather than with crystalline surfaces^33^.

While the closest homologs of EngU also have an inserted CBM4 domain between stand β-4 and helix α-4 of the predicted (β/α)_8_ barrel, other sequences have a shorter insertion at the same place, but with no detectable similarity to CBM4 (for example ORF Igag_0510 of *Ignisphaera aggregans*, ADM27348.1 or ORF B2J77_11410 of *Pseudomonas parafulva*, AQW68778.1). Thus, different members of GH148 carry different insertions at identical positions of their catalytic domain. Interestingly, in the available databases we could not find any proteins belonging to the new GH148 family that lacked an inserted sequence between the two parts (A1 and A2) of the catalytic domain. The reason for this is currently unknown but it can be speculated that apparently the inserted additional domains in GH148 generally play important roles for the enzymes’ physiological function(s).

GH-A clan proteins exhibit a (β/α)_8_ TIM barrel structure with two catalytic acidic residues, found at the C-terminal ends of β-strands 4 and 7^34^. Secondary structure-based amino acid sequence alignment of EngU with representative enzymes of the GH-A clan with known structure showed that the GH-A1 and GH-A2 parts of EngU correspond to the two halves of the (β/α)_8_ barrel, with the CBM module placed after the fourth β strand (Supplementary Fig. 1). This split-barrel structure is reminiscent of the GH42 β-galactosidase from *Thermus thermophilus* A4 where there is an extra region inserted between β-4 and α-4, termed subdomain H, which has been implicated in forming the active-site pocket through trimerization^35^. In EngU however, a whole functional module is inserted at this position, separating the two parts of the TIM barrel by approx. 250 amino acid residues (Fig. 6). It has been shown for the TIM barrel enzyme HisF from *Thermotoga maritima* that the two halves of the barrel, when co-expressed *in vivo* or allowed to refold together *in vitro*, can assemble into a functional (β/α)_8_ barrel^36^. This protein, which has a completely different physiological function (histidine biosynthesis) than EngU displays an internal twofold repeat pattern and has provided evidence for the evolution of β/α barrels from an ancestral half-barrel^37^. Interestingly, a weak but still detectable internal sequence repeat pattern could also be found in EngU, when the two predicted half-barrels were compared to each other (15% identity, Supplementary Fig. 2). Similar to HisF, when we expressed individually but simultaneously the two parts of the barrel in the cell, the glucanase activity could be restored. Thus, although the 3D-structure of EngU is not yet available, this metagenomic glucanase provides additional support for the mode of evolution of (β/α)_8_ barrels proposed by Lang *et al*. for a different class of enzymes^37^.

Enzymes from clan GH-A share a retaining mechanism of glycosidic bond cleavage involving catalytic glutamic acid residues. Substitution of the two glutamates E239 (INNEN) and E581 (VTEYN) with the smaller nonpolar amino acid alanine led to a complete loss of EngU activity towards β-glucan (Table 4). We propose that E239 represents the acid/base residue and E581 is the nucleophile residue based on the predicted catalytic residues in A4-β-Gal of *Thermus thermophilus* ^35^. The significant activity loss of the EngU D123A mutant (3% residual activity) may be due to the close vicinity of D123 to a structurally or catalytically crucial amino acid such as Arg62 of the clan GH-A family GH5^38^.

Although EngU showed local, weak similarity to the GH42 family, no biochemical characteristics of a β-galactosidase were observed, as X-Gal or lactose could not be hydrolyzed. EngU was most active towards mixed-linkage β-1,3/β-1,4-substrates such as barley β-glucan and lichenin. Only low activity was found with uniformly β-1,4-linked cellulosic substrates such as CMC and HEC. Other polymers containing β-1,3-glycosidic bonds only (zymosan, curdlan, auxoferm and pachyman) or mixed β-1,3-1,6 bonds (laminarin) were not hydrolyzed by EngU.

β-1,3- and β-1,4-glucanases are differentiated according to their mode of action (*endo*- or *exo*-), the type of substrate hydrolyzed and the scissile linkage preference^39^. The specificity of an *endo-*cleaving enzyme from this group is assigned to an EC entry such as 3.2.1.4 (*endo*-1,4-β-glucanase), 3.2.1.6 (laminarinase, includes enzymes hydrolyzing β-1,3 or β-1,4 linkages in C-3 substituted glucopyranose units, as in laminarin, lichenin and cereal β-glucans), 3.2.1.39 (*endo*-1,3-β-glucanases which act on β-1,3-glucosidic linkages in β-1,3-glucans, e.g., laminarin and pachyman) and EC 3.2.1.73 (lichenase, the enzymes act on β-1,4 linkages in β-glucans containing β-1,3 and β-1,4 bonds, e.g., lichenin and cereal β-glucans). Our results lead to the conclusion that EngU is an *endo*-β-glucanase/lichenase with a unique scissile linkage preference for β-1,3 linkages, which does not allow an unambiguous assignment to an existing Enzyme Commission group.

The analysis of the β-glucan hydrolysis products liberated by EngU over time shows an *endo*-type cleavage of the polysaccharide, and subsequent cleavage of the long oligosaccharide intermediates leads to cellotriose as the main final product. EngU cleaves predominantly β-1,3 glycosidic bonds which are adjacent to a β-1,4 bond (Fig. 6), as was shown by analysis of the products formed from β-glucan and from the mixed-linkage oligosaccharides G_4__b_ and G_4__c_ (Fig. 5). This cleavage pattern is distinct from all other lichenases characterized so far, which hydrolyze mainly the β-1,4 linkages of C-3 substituted glucose units in mixed-linkage β-1,4/β-1,3-glucans. EngU can also weakly cleave β-1,4-glycosidic bonds which is evident from the weak activity with cellopentaose, cellotriose, CMC and HEC (Table 2 and Fig. 5). Presumably a trisaccharide is the smallest unit that the enzyme can process, because the disaccharide products were not degraded further. According to the CAZy database, lichenase (EC 3.2.1.73) activities can be found in 9 different GH families, which do not share significant primary structure similarity. In plants, *endo*-β-1,3/β-1,4-glucanases are represented by GH families 12 and 17, while these enzymes from the bacteria are mostly members of GH5 and GH16.

In conclusion, EngU is the first characterized representative of a new type of β-1,3/β-1,4-glucanase displaying a novel scissile linkage preference and represents the first characterized member of a new GH family within the GH-A clan. Highly unusual, the enzyme carries a CBM4-type carbohydrate-binding module inserted between the catalytic glutamate residues of its catalytic module. Also, the enzyme carries a C-terminal part without similarity to other known modules, whose role awaits further clarification but which appears to be necessary for functional expression of the enzyme. The unusual mosaic architecture of EngU and related enzymes demonstrates that the *in silico* detection and function prediction of such proteins with the current bioinformatics tools is challenging. Given the broad distribution and multitude of enzyme activities of TIM barrel proteins it will be interesting to investigate if similar patchy structures can be found in other TIM barrel enzymes.

## Methods

### Bacterial strains and vectors

*Escherichia coli* EPI300-T1 (Epicentre, Madison, USA) was used for cultivation of the pCC1FOS large-insert fosmid clone FosK48 H3 which was obtained from the Avachinsky crater metagenomic library^22^. The *E*. *coli* strains XL1-Blue (Stratagene, La Jolla, USA), DH10B and TOP10 (Invitrogen, Carlsbad, USA) were used for general cloning purposes, propagation of recombinant plasmids and cloning. The *E*. *coli* strain BL21 (DE3) was used as a host for protein expression from pETDuet and pGEX-4T-2 vectors (Merck Millipore, Darmstadt, Germany). Bacterial cultures were grown at 37 °C in Lysogeny Broth (LB) or LB agar plates supplemented with the appropriate antibiotics. Ampicillin was used at 100 µg/ml, kanamycin at 25 µg/ml and chloramphenicol at 12.5 µg/ml.

### Bioinformatic analysis

Potential signal peptides were predicted using the signalP software^40^, blastP^41,42^ and the *nr* database were used for the detection of putative EngU homologs. The Pfam database (version 30)^43^ and the HMMER program (version 3.1)^44^ were used to generate sequence alignments of EngU with selected Pfam families. Secondary structure-based alignments were performed with T-Coffee^45^ and ENDScript^46^.

### Construction of plasmids for the expression of wild type and truncated EngU variants and mutants

The DNA fragments encoding EngU were amplified by PCR with *Pfu* DNA polymerase (ThermoFisher Scientific) or with Q5 polymerase (New England BioLabs) according to the manufacturer’s instructions. The PCR products for e*ngU* were cloned using the Champion™ pET101 directional TOPO® Expression Kit (Invitrogen). The PCR product generated with primers 48H1.F and 48H1.R encodes the full-length EngU protein with its predicted signal peptide at the N-terminus. Truncated versions lacking the signal peptide were obtained with 48H2.F as the forward primer. The pETDuet-1 vector was used to express either one or two target ORFs by means of its two multiple cloning sites, MCS1 and MCS2. EngU fragments were cloned into MCS1 of pETDuet *via* NcoI and HindIII and into MCS2 *via* NdeI and KpnI. Plasmid construction was done either by restriction and ligation, using T4 DNA ligase (ThermoFisher Scientific) or by using Gibson assembly (Gibson Assembly Kit, New England Labs). The plasmid pGST-CBM, used for the expression of CBM4 part of EngU (as a N-terminal GST fusion protein, termed EngU_GST-CBM_), was constructed by amplification by PCR of the DNA corresponding to amino acid positions 292–473 of EngU and cloning of the PCR product in the BamHI site of the pGEX-4T-2 vector (Thermo Fischer Scientific) *via* Gibson assembly.

The ChangeIT site directed mutagenesis kit (Affymetrix) was used to generate all eight EngU mutants (EngU D123A, D151A, E183A, E235A, E239A, E581A, E593A and E605A). The mutations were verified by sequencing. All primers used in the cloning and mutagenesis steps are listed in Supplementary Table 1.

### Expression and purification of recombinant EngU and of EngU_GST-CBM_ fusion protein

*E*. *coli* BL21 cultures (500 ml) were grown in Erlenmeyer flasks to an optical density of 0.6–0.7 (600 nm) before induction of protein expression with 1 mM isopropyl-β-d-thiogalactopyranoside (IPTG). After 12 hours at 37 °C, the cells were harvested by centrifugation, resuspended in 50 mM phosphate buffer (pH 7) and disrupted in a French pressure cell (SLM Aminco, Urbana, USA) or by sonication (Hielscher sonicator, amplitude 50%, interval 0.4 at 4 °C). After removal of the cell debris by centrifugation at 20000 *g* for 15 min, the BL21 EngU lysate was subjected to heat treatment at 85 °C for 20 min. The supernatant containing the thermostable and soluble protein was further purified using an Äkta FPLC (GE Healthcare, UK) equipped with a Source 15S cation exchange column (4.6/100PE), using a linear gradient from 0 to 1 M NaCl. SDS-PAGE^47^ was used to monitor the protein purification steps and to analyze protein purity and integrity.

The EngU_GST-CBM_ as well as the GST proteins were purified from crude extracts of BL21 cells transformed with the respective plasmids by using Glutathione Sepharose 4B gravity flow columns (Thermo Fischer Scientific), followed by a gel filtration strep on a Superdex200 column using 50 mM Tris pH 8.0, 150 mM NaCl as the buffer.

### Enzyme assays

The optimal reaction conditions for EngU were tested at different temperatures in an oil bath rotary shaker (Infors HT Aquatron, Bottmingen, Switzerland). The pH optimum was determined using (all at 50 mM) McIlvaine buffer (pH 4–6), phosphate buffer (pH 5.5–8) and Tris-HCl buffer (pH 8–10). Resistance against thermoinactivation was determined by incubation of the enzyme (0.2 mg/mL) in 50 mM McIlvaine buffer pH 6 at temperatures between 60 °C and 97 °C. The enzymatic activity against polysaccharide substrates was determined using the 3,5-dinitrosalicylic acid (DNS) colorimetric assay^48^. One unit of enzyme activity corresponds to 1 µmole of reducing sugar ends released from the substrate per minute (for tested substrates see Table 2). Assay mixtures contained 250 µl of 1% (w/v) substrate, purified protein sample and 100 µL McIlvaine buffer (pH 6) in a total volume of 500 µl and were incubated for 10 min at 90 °C. The reaction was stopped by adding 750 µL DNS reagent followed by incubation at 95 °C for 5 min. The absorbance at 575 nm was measured in a spectrophotometer and the specific activity was calculated using a calibration curve prepared with glucose as a standard. Protein concentration in the samples was determined with the Bradford reagent, using bovine serum albumin as a standard.

### Analysis of binding of EngU_GST-CBM_ to soluble and insoluble substrates

The polysaccharide binding properties of the carbohydrate binding module of EngU (expressed as a N-terminal GST fusion protein, EngU_GST-CBM_) were investigated with affinity gel electrophoresis for the soluble substrates which were included into native PAGE gels, and with batch binding assays for the insoluble substrates. The batch binding assays were performed in 5 mM Tris HCl containing 0.2 M NaCl at 4 °C. The reactions (1 ml), containing 100 µg purified EngU_GST-CBM_ or GST and 50 mg insoluble substrate, were incubated on a shaker, samples were taken at the indicated time points (Supplementary Figure 4) and centrifuged for 2 min at 20 000 *g* to remove the ligand together with the bound protein. The amount of free protein in the supernatant was determined with the micro BCA Protein Assay Kit (Thermo Fischer Scientific). The measurements were performed in triplicate, using purified GST as a non-binding control. Affinity gel electrophoresis (6% polyacrylamide) with and without soluble substrates was performed as described by Zverlov *et al*.^49^. Bovine serum albumin (BSA) and GST were used as non-interacting negative controls. The relative migration distances with (*r*) and without (*r*_0_) substrate were calculated as the ratio of the migration distance of the major protein band and the migration front of the gel. The retardation coefficient *K*r was determined from the relative migration distances: $K{\rm{r}}=\frac{r}{r0-r}\times [{\rm{S}}]$, where [S] is the substrate concentration (mg × ml^−1^). Subtrate concentrations from 0 to 1 mg/ml were used in the affinity gel electrophoresis experiments.

### Analysis of glucan and oligosaccharide degradation products by TLC and HPAEC-PAD

Degradation products of enzymatic hydrolysis of barley β-glucan (low viscosity), cellopentaose (G_5_), cellotriose (G_3_), and the mixed linkage β-gluco-oligosaccharides glucotetraose B (also abbreviated as G_4b_, G4G4G3G) and glucotetraose C (also abbreviated as G_4c_, G4G3G4G) (each glucose moiety abbreviated as G; the reducing end is the rightmost G; the numbers 4 and 3 represent β-1,4- and β-1,3-glucosidic linkages, respectively), all purchased from Megazyme (Megazyme International, Ireland), were analyzed by thin layer chromatography on silica gel plates (type 60 F254, Merck, Darmstadt, Germany) and by HPAEC-PAD (High performance anion exchange chromatography with pulsed amperometric detection). For the reactions (100 µl), 0.5% barley β-glucan or 0.1% oligosaccharide was mixed with purified EngU (1.5 µg) or LicB (0.7 µg) in MES buffer pH 5.5 and incubated at 80 °C for EngU or 60 °C for LicB. The standard mix used for identifying the hydrolysis products consisted of 0.1% (w/v) of glucose, cellobiose, cellotriose, cellotetraose, cellopentaose and cellohexaose (G_1–6_) in MES buffer pH 5.5. The mobile phase used for the TLC was butanol/ethanol/water (5:5:4 v/v/v) or acetonitrile/water (8:2 v/v). After separation the sugars were visualized by spraying the plate with a solution of 1% (w/v) diphenylamine and 1% (v/v) aniline in acetone, followed by heating at 120 °C for 10 min. The analysis of oligosaccharides by HPAEC-PAD was performed with an ICS-3000 Dionex system equipped with a CarboPac PA1 column (4 × 250 mm) and a PA1 pre-column (4 × 50 mm). The reactions were diluted tenfold, 25 µL were injected and the analysis was performed at 30 °C at a flow rate of 1 mL/min. Separation was achieved in 100 mM sodium hydroxide using an eluent gradient profile based on sodium acetate (0–45 min: a linear gradient from 0 to 100 mM sodium acetate; 45–47 min: a linear gradient from 100 to 500 mM sodium acetate; 47–49 min: 500 mM sodium acetate; 49–50 min: a linear gradient from 500 to 0 mM sodium acetate; 50–60 min: 0 mM sodium acetate). Detection was done using the carbohydrate standard quad waveform.

### Accession codes

Nucleotide and amino acid sequence data for EngU are available in the DDBJ/EMBL/GenBank databases under the accession number LT622840.

### Data availability

All data generated or analysed during this study are included in this published article (and its Supplementary Information files).

## Electronic supplementary material

supplementary file
