# Supplementary material for: A metagenome-derived thermostable β-glucanase with an unusual module architecture which defines the new glycoside hydrolase family GH148
Source: Sci Rep. 2017 Dec 11;7:17306. doi: 10.1038/s41598-017-16839-8 (PMC5725463; doi:10.1038/s41598-017-16839-8)

Supplementary material

A metagenome-derived thermostable β-glucanase with an unusual module architecture which defines the new glycoside hydrolase family GHxyz

Angel Angelov[1+], Vu Thuy Trang Pham[1+], Maria Übelacker[1], Silja Brady[2], Benedikt Leis[1], Nicole Pill[1], Judith Brolle[1], Matthias Mechelke[1], Matthias Mörch[1], Bernard Henrissat[3], and Wolfgang Liebl[1*]

[1]Department of Microbiology, School of Life Sciences Weihenstephan, Technical University of Munich, Freising-Weihenstephan, Germany

[2]Department of Genomic and Applied Microbiology and Göttingen Genomics Laboratory, Georg-August University Göttingen, Göttingen, Germany

[3]Architecture et Function des Macromolécules Biologiques, CNRS, Aix-Marseille University, Marseille, France.

Supplementary Table S1. Names and sequence of the oligonucleotides used in the study.

Primername	Sequence
Bgl_for	GATCCATATGCGTTGCGGTCTGACACAGGAA
Bgl_rev	GATCAAGCTTTCATTGCACCACAACTTCATAC
cbm_rev	GATCAAGCTTTCACGTCTGTGCCCCCAAGTTGCTG
gh42_rev	GATCAAGCTTTCAAAAGTTGGCGTTGCGCAACAT
Bgl_for ecoRI	GATCGAATTCATGCGTTGCGGTCTGACACA
Bgl_for NcoI	GATGCCATGGGTTGCGGTCTGACACA
cterm_for	GATCCATATGGCACGGGTCATATTCAG
Bgl_rev KpnI	GGCCGGTACCTTTCATTGCACCACAACTTCAT
Duet_Eng48 F1	TAACTTTAAGAAGGAGATATACCATGCGTTGCGGTCTGAC
Duet_Eng48 R	CTTAAGCATTATGCGGCCGCATCATTGCACCACAACTTCA
gh42a_for	ACTTTAAGAAGGAGATATACCTGGACTACCTGATTGCCCA
gh42 b rev2	ATTATGCGGCCGCAGTGTCACCTGACAACCACTTGACGCT
Duet Eng48 F1	TAACTTTAAGAAGGAGATATACCATGCGTTGCGGTCTGAC
Duet Eng48 R	CTTAAGCATTATGCGGCCGCATCATTGCACCACAACTTCA
E239A	CAAGCGTGAACGAGACCGTTCGCGTTGTTGATTTCCACAAAG
E581A	GTAAACCCTTCTCGGTTACAGCGTACAACCATTCTGCTCCG
D151A_rev	AGTTGGGCAATCAGGTACGCCAGTCGGTCAAGGGCTT
E183A_for	GCCGACGGTTTGCCCAAAGCGATTGAACAACTTGGTT
D123A R	CGTTGGGAAACTCTTGCATCGCCATGTGATGGAATCGGAC
E235A R	CCGTTTTCGTTGTTGATCGCCACAAAGGCAATTGCAGGGT
E593A R	CGAGCAACAAGAAGGCCGCACTGCTGTAAGTGTT
D605A R	GAAGGCGTAAATTGCGTCCCACGCCTGAAGGGCTGCGTAG
pET-Duet 1 SEQ	TGGCAAGTGTAGCGGTCACG
pET Upstream	ATGCGTCCGGCGTAGA
Duet DOWN1	GATTATGCGGCCGTGTACAA
DuetUP2 Primer	TTGTACACGGCCGCATAATC
CBM-292	ATCGGATCTGGTTCCGCGTGGATCCGGCGAAGAGATGTTGCGCAAC
CBM-473	GTCGACCCGGGAATTCCTGGGTCACGTTCGTTCTCCAAAACAAC

Legends to supplementary figures

Supplementary figure 1.

Modular organization of EngU and structure-based alignment of EngU and selected GH42 enzymes with known structures. The alignment was performed with T-Coffee[1] and the proteins used in the alignment are 1KWG (*Thermus thermophilus* A4 β-galactosidase), 3TTS (*Bacillus circulans* β-galactosidase), 4OIF (*Geobacillus stearothermophilus* β-galactosidase), 4UNI (*Bifidobacterium animalis* subsp. *lactis* β-(1,6)-galactosidase) and 4UZS (*Bifidobacterium bifidum* β-galactosidase). The catalytic residues are marked with a red triangle in the alignment and with a red line in the EngU scheme. The numbering above the alignment is relative to 1KWG.

Supplementary figure 2

Alignment of the two predicted half-barrels of EngU to each other. The numbers on the left correspond to coordinates of the 905 amino acids full-length EngU protein. The pairwise alignment was performed with the Needle program from the EMBOSS package[2].

Supplementary figure 3

HPAEC-PAD analysis of the end products released from barley β-glucan by EngU (A) and LicB (B), from the glucotetraoses G_{4b} (C), G_{4c} (D) by EngU and from cellopentaose by EngU (E). The grey line represents a control experiment without enzyme. The black line is a chromatogram of a mixture of glucose (G), cellobiose (G_2), cellotriose (G_3), cellotetraose(G_4), cellopentaose (G_5) and cellohexaose (G_6).

Supplementary figure 4

Binding properties of recombinant $EngU_{GST-CBM}$

References to supplementary material

1. Di Tommaso, P., Moretti, S., Xenarios, I., Orobitg, M., Montanyola, A., Chang, J. M., Taly, J. F., and Notredame, C. (2011) T-Coffee: A web server for the multiple sequence alignment of protein and RNA sequences using structural information and homology extension. *Nucleic Acids Res.* **39**, 13–17

2. Li, W., Cowley, A., Uludag, M., Gur, T., McWilliam, H., Squizzato, S., Park, Y. M., Buso, N., and Lopez, R. (2015) The EMBL-EBI bioinformatics web and programmatic tools framework. *Nucleic Acids Res.* **43**, W580–W584

Supplementary figure 1

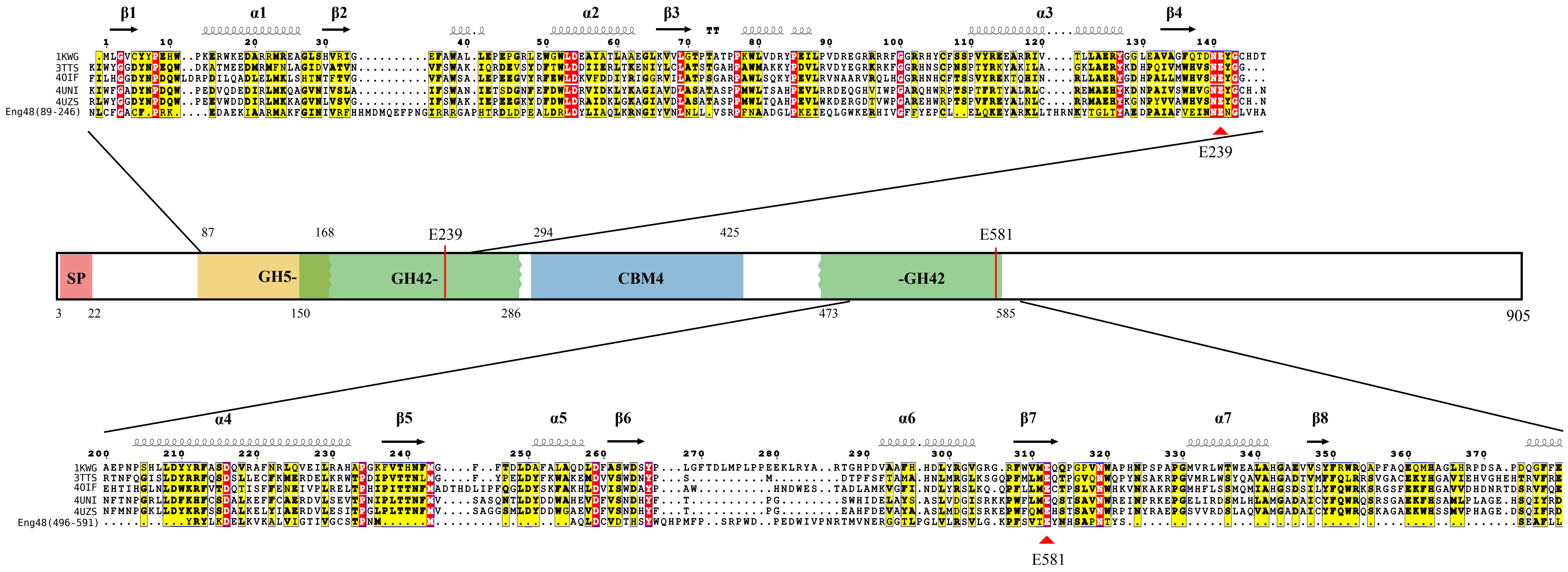

Supplementary figure 2

```
              1                   11                  21                  31                  41
Conservation                  *   :   :   . * *   .   .   : * : :                     :   : * *           : . :
80-290        K Q R I R F L G V N L C F G A C F P R - K E D A E K I A A R M A K F G I N I V R F H H M D M Q E F P
461-620       - - - - - - - - V P I F L R G C F G E R T P E A Q R - - - - - - - - - - D W I R F L W E T E N R Y W

              51                  61                  71                  81                  91
Conservation  :   *   *               :                               * :               : : * * * .       .
80-290        N G I R R R G A P H T R D - - - - - - - - - - - - - - L D P E A L D R L D Y L I A Q L K R - - N G I
461-620       Q T I Y R Y L K D - - - E L K V K A L V I G T I V G C S T P N - - - - - - - M M A Q L D C V D T H S

              101                 111                 121                 131                 141
Conservation  *   :     : :   * * * : : .   *   :     :       : .   :       :   * : .   . .   *       *   : :
80-290        Y V N L N L L V S R P F N A A D G L P K E I E Q L G W K E R H I V G F F Y E P C L E L Q K E Y A R K
461-620       Y W Q H P M F P S R P W D P E D W I V P N R T M V N E R G G T L P G L V L R S V L - - G K P F S - -

              151                 161                 171                 181                 191
Conservation    : * .   * :   :     * * : . : .   :   : .
80-290        L L T H R N K Y T G L T Y A E D P A I A F V E I N N E N G L V H A W L G N D I D N M P K V F L D E L
461-620       - V T E Y N H S A P N T Y S S E A F L L L A - - - A Y - - - - - - - - - - - - - - - - - - - - - - -

              201                 211                 221
Conservation          : : *     *   * .   :   .   * :
80-290        Q R Q W N E W L R A R Y G T T E K L R K A W G V R E E P
461-620       - A A L Q D W - D A I Y A F S Y S H R R D - - - - - - -
```

Supplementary figure 3

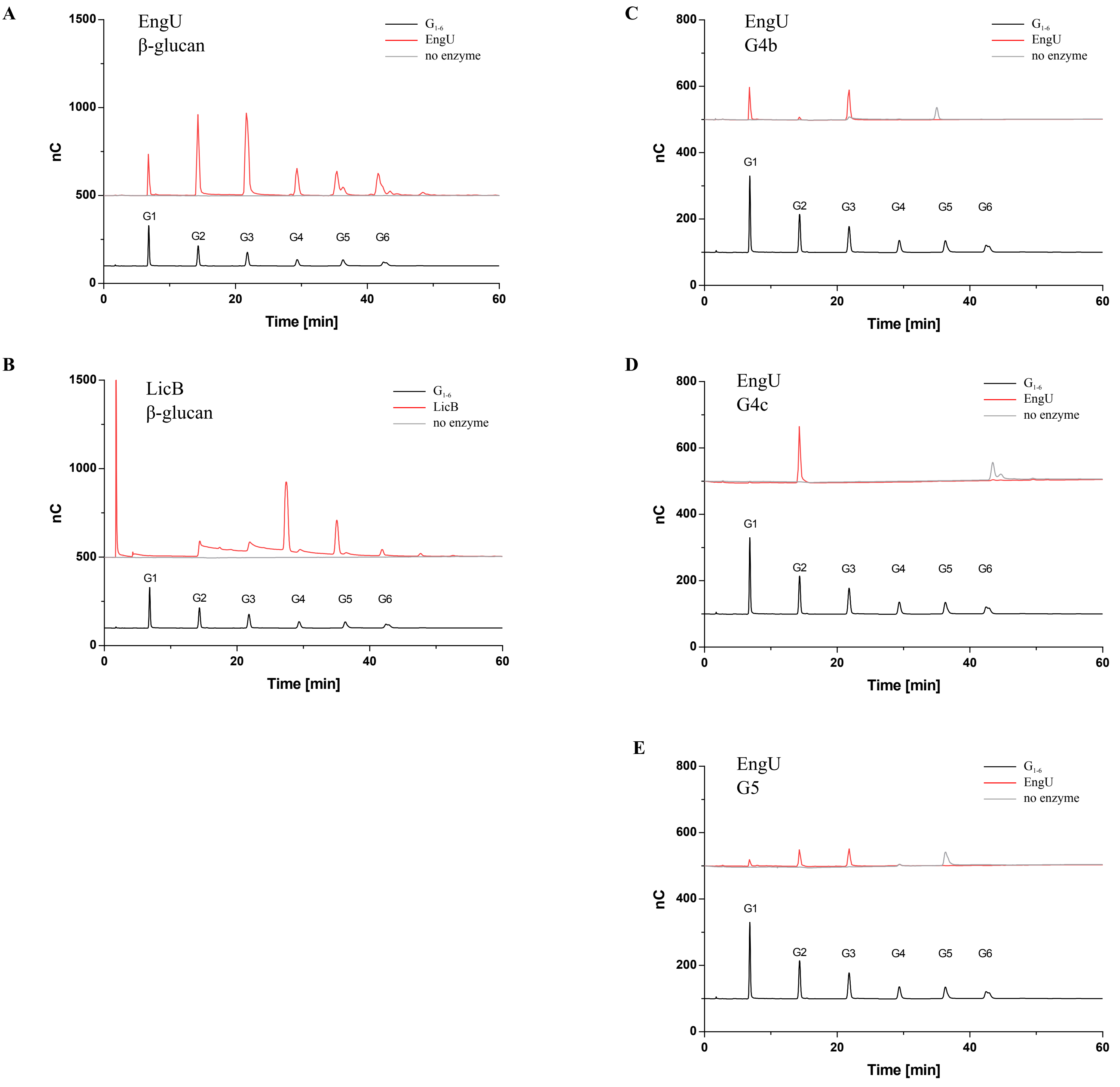

Supplementary figure 4. Binding properties of recombinant $EngU_{GST-CBM}$. The purified GST, $EngU_{GST-CBM}$ and bovine serum albumin (BSA) were used in gel retardation assays with soluble substrates (A) and in batch binding assays with insoluble substrates (B).

A. An example of a gel reatardation experiment of $EngU_{GST-CBM}$ with β-glucan and laminarin. The native PAGE gels were run without (-) or with (+) 0.1 % (w/v) substrate included in the gels (affinity gel electrophoresis). The relative migration distances *r0* (without substrate) and *r* (with substrate) were determined as the ratio of the migration distance of the major protein band and the migration front of the gel.

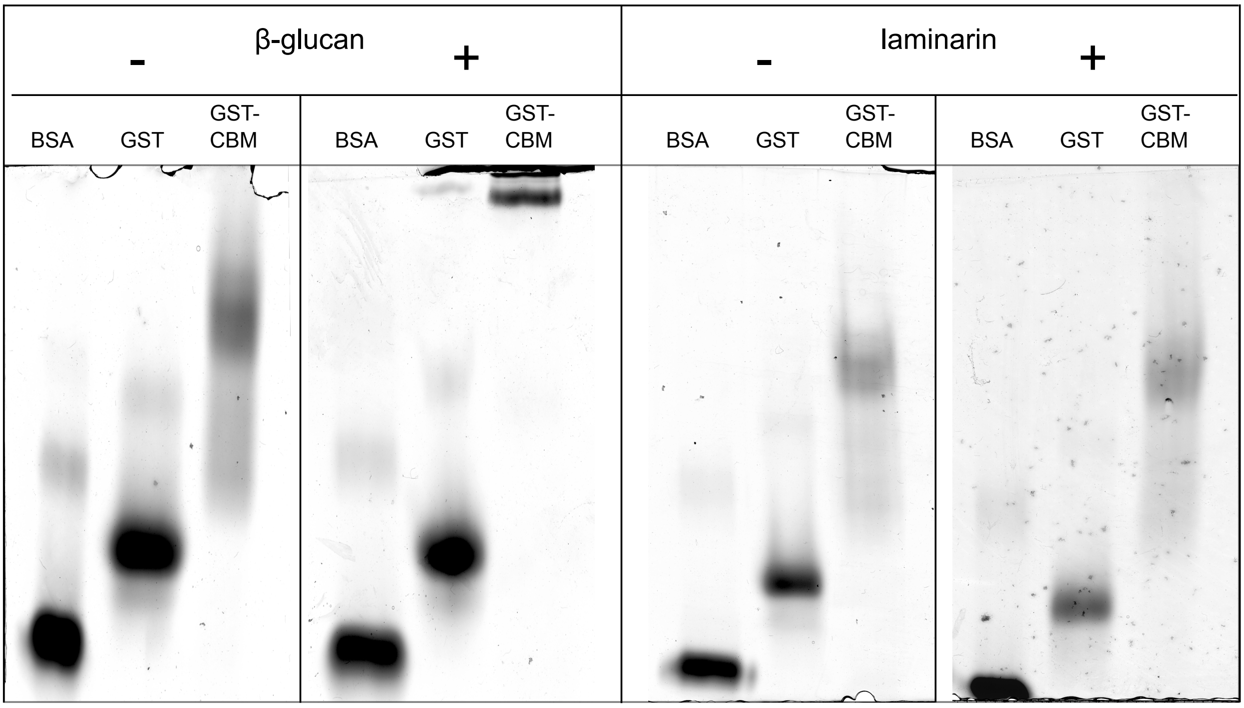

B. Kinetics of binding of purified $EngU_{GST-CBM}$ in batch assays to cellulose preparations with increasing level of crystallinity (top row) and to pachyman, Auxoferm and birch wood xylan (bottom row). Three replicate measurements for each time point were performed, (grey dots). The y-axis represents the residual protein concentration (in % relative to the initial) in the cleared supernatants after incubation with the substrate.

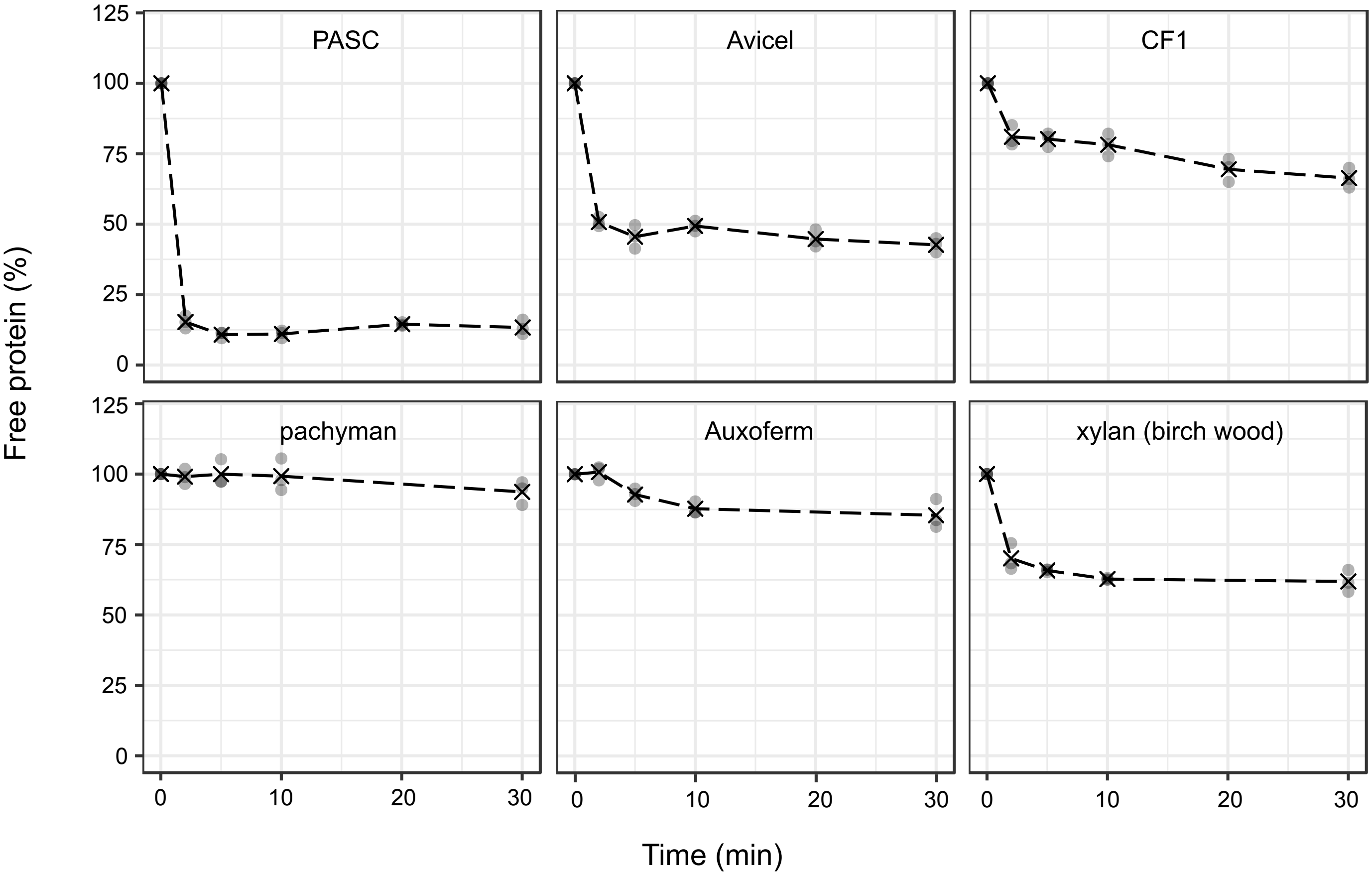

Supplement: Supplementary file 1 — supplementary file [file 41598_2017_16839_MOESM1_ESM.pdf]
